# Behaviours That Challenge in Special Schools: Heterogeneity of Profiles Among Students With Intellectual and Developmental Disabilities and Implications for Educational Support

**DOI:** 10.1111/jir.70155

**Published:** 2026-08-02

**Authors:** Zakaria Mestari, Severine Pradel, Aline Veyre, Rachel Sermier Dessemontet

**Affiliations:** ^1^ University of Teacher Education of State of Vaud Lausanne Switzerland; ^2^ University of Applied Sciences and Arts Western Lausanne Switzerland

**Keywords:** challenging behaviours, developmental disabilities, multilevel latent class analysis, positive behaviour support

## Abstract

**Background:**

This study aimed to examine the types and frequency of challenging behaviours (CB) among students with intellectual and developmental disabilities in special education schools and identify profiles among them.

**Methods:**

Data were collected from 239 special school staff members, who reported information on 635 students via an online survey. A multilevel latent class analysis was used to identify subgroups of students based on the type and frequency of CB while controlling for respondent nesting.

**Results:**

According to respondents, among students in their classrooms (*M* = 6.02, SD = 2.54) about half displayed persistent CB (*M* = 2.88, SD = 2.08). Oppositional and socially inappropriate behaviours were the most frequently reported, displayed by over 90% of students with CB and occurring daily. Group 1 (*n* = 169, *M* = 11.00 years) exhibited the most severe and frequent CB across nearly all categories, mainly physical and verbal aggression and destructive behaviours. Group 2 (*n* = 200, *M* = 9.90 years) showed high levels of physical aggression, self‐injurious and destructive behaviours, but very low levels of verbal aggression. Group 3 (*n* = 131, *M* = 12.17 years) was characterised by overall low levels of CB, with some verbal aggression. Finally, Group 4 (*n* = 135, *M* = 10.86 years) showed elevated rates of destructive behaviours, a moderate prevalence of physical aggression and self‐injurious behaviours, in addition to the lowest rate of socially inappropriate behaviours.

**Conclusions:**

These profiles highlight the heterogeneity of CB presentations among students in specialised educational settings and underscore the need for tailored educational and behavioural support and intervention.

## 1 Introduction

Challenging behaviours (CB) are defined as behaviours whose frequency, duration or intensity pose a risk to the individual or others, restrict access to educational or community settings, interfere with the development of positive relationships and age‐appropriate skills, and have detrimental effects on the well‐being and quality of life of the individual and those around them (Dunlap et al. 2018; Emerson 2001). Examples of CB include physical and verbal aggression, self‐injurious behaviour, property destruction, oppositional behaviour and socially inappropriate behaviour (Newcomb and Hagopian 2018). However, the impact of these behaviours on the individual and those around them remains crucial for distinguishing disturbing behaviours from CB (e.g., refusing to complete a task once or twice a week vs. refusing to complete several tasks multiple times daily).

About one‐third of school‐age children are expected to experience emotional or behavioural difficulties during their education (Forness et al. 2012). Students with intellectual and developmental disabilities (IDD) are at significantly higher risk (Hastings et al. 2013), since CB are notably more prevalent among them compared to their peers without a diagnosis (Fodstad et al. 2012; Korbut et al. 2020; McClintock et al. 2003). IDD includes conditions such as intellectual disability and autism, which emerge early in life, affect functioning across multiple domains and persist throughout life (APA 2022). Studies report CB in 48% to 60% of students with intellectual disabilities (Simó‐Pinatella et al. 2019) and 36% to 48% of students with autism (Holden and Gitlesen 2006; Mutluer et al. 2022), though some estimates exceed 90% (Anixt et al. 2020; Jang et al. 2011). CB such as aggression and self‐injury are more common among individuals with intellectual disability who also have autism and among persons with intellectual disability who have deficits in communication (McClintock et al. 2003). Nevertheless, CB in persons with IDD are the product of dynamic interactions between a complex set of biological, psychosocial and environmental factors (Bowring et al. 2019; Hastings et al. 2013). Importantly, CB in this population are highly heterogeneous in type, frequency and intensity, encompassing a wide range of behavioural profiles (Bowring et al. 2016; Reyes‐Martín et al. 2022). Recognising and distinguishing these profiles is therefore essential for implementing tailored strategies and evidence‐based interventions.

The presence of CB has consequences for students' well‐being, learning and social participation by reducing opportunities for socialisation and increasing the risk of school exclusion (Lloyd and Kennedy 2014; O'Grady and Ostrosky 2021), and may, later in life, lead to restrictive practices such as physical restraint or medication (Büschi et al. 2021). These behaviours are also an important source of family stress, affecting caregivers' quality of life, psychological health and social inclusion (Mello et al. 2022; Mitter et al. 2019; Smart et al. 2023). Teachers and school staff are similarly impacted, with 31% to 52% of students with IDD in special schools reported to exhibit CB (Cormack et al. 2000; Molteno et al. 2001; Müller et al. 2020). These behaviours not only compromise student inclusion and safety but are also a key contributor to staff stress, injury, burnout and turnover (Gilmour and Wehby 2020; Nistor and Chilin 2013). This highlights the need for socially valid and evidence‐based interventions, as the implementation of such interventions in schools is essential to mitigate the risks associated with CB and to support students' long‐term educational and social outcomes (Reyes‐Martín et al. 2022).

Positive Behaviour Support (PBS) is a well‐established, evidence‐based, multicomponent and multitiered framework that promotes proactive, non‐aversive and person‐centred approaches to reducing CB through close collaboration with key stakeholders (Cooper et al. 2020; Gore et al. 2013; Kincaid 2018). Within school settings, PBS is typically operationalised through a tiered system of supports to address student well‐being and behaviours using evidence‐based practices (Beqiraj et al. 2022; Corcoran and Edward Thomas 2021). Tier 1 provides universal strategies for all students to promote appropriate and functional behaviours and prevent CB. Tier 2 offers targeted interventions for students for whom Tier 1 supports are insufficient. Tier 3 delivers intensive, individualised support for students with persistent or more severe needs (Simonsen et al. 2021). At the individual level (Tier 3), effective PBS interventions rely on functional behaviour assessment, the development of individualised intervention plans that integrate preventive strategies and explicit instruction of alternative behaviours and ongoing data‐based decision‐making (Dunlap et al. 2018; Walker et al. 2017). Most studies on the implementation of PBS for students with IDD in special education settings focus on individualised interventions (Tier 3), rather than on school‐wide PBS (Beqiraj et al. 2022). Such individualised interventions are efficient in reducing CB and increasing alternative behaviours among students with IDD either in inclusive or specialised school settings (Goh and Bambara 2010; Lory et al. 2020; Walker et al. 2017).

In Switzerland, where this study was conducted, inclusive schooling and special schools for students with IDD exist in parallel. Many students with IDD, especially those with more severe limitations in adaptive behaviour and intellectual functioning attend special schools (Müller et al. 2020; Sermier Dessemontet et al. 2012). Importantly, a growing body of research demonstrates that students with moderate and severe IDD possess substantial learning potential and a strong capacity for meaningful progress. When evidence‐based educational and behavioural supports are implemented, these students can achieve significant gains both academically (e.g., Sermier Dessemontet et al. 2019; Spooner et al. 2019) and behaviourally (e.g., Corr et al. 2025; LaVigna and Willis 2012), underscoring the importance of high expectations and proactive support approaches.

To date, research on the prevalence and characteristics of CB in Switzerland's special schools is still limited. Existing studies have largely focused on an adult population (Büschi and Lustenberger 2019; Lustenberger et al. 2019) or on staff perceptions of student stress (Amstad and Müller 2020). For instance, Lustenberger et al. (2019) reported a 28% prevalence of CB in adults with IDD in institutions, with aggression, motor agitation and self‐injury being the most common. Müller et al. (2020) found that 51.8% of students in special schools displayed high levels of CB. While these studies offer valuable insights, empirical data on the specific nature and patterns of CB in this specific context remain scarce. Beyond prevalence and frequency, the present study seeks to identify and describe distinct CB profiles among students with IDD to contribute a more nuanced understanding of their behavioural support needs.

## 2 Objectives

The present study is part of a broader project aimed at implementing PBS in special schools within the French‐speaking regions of Switzerland. The project is structured according to the stages of the ORBIT model for behavioural interventions (Czajkowski et al. 2015). During Phase I (intervention design), a comprehensive needs assessment was conducted to document the characteristics of CB among students with IDD in special schools, as well as teaching staff's understanding, strategies and training needs related to CB. The Prevent‐Teach‐Reinforce model, a Tier‐3 PBS programme, was translated and adapted through consultations with experts and stakeholder focus groups. Phase II (preliminary testing) involves piloting the adapted programme through a series of single‐case proof‐of‐concept studies, followed by a pilot randomised feasibility study using a waitlist‐control design.

The present article reports on the needs assessment component of ORBIT Phase I and seeks to enhance our understanding of the types and frequency of CB exhibited by students with IDD in special school settings. Specifically, the study addresses the following research questions:

1. What types of CB are displayed by students within special schools, and how frequently do they occur according to staff?
2. What distinct profiles can be identified among students with IDD, considering the type and frequency of CB?
3. To what extent are these profiles distinct regarding age, diagnoses and communication skills?

## 3 Methods

### 3.1 Design

This cross‐sectional study used an online survey to collect the data required to address the three research questions. Due to its survey‐based design, the present study was reviewed and deemed exempt from ethics approval by the Swiss Commission for Ethics in Human Research of the Canton of Vaud (reference number: Req‐2024‐00420). A formal authorisation was subsequently sought and obtained by contacting the departments or offices responsible for special education across six French‐speaking cantons in Switzerland. Once approval to conduct the study was granted in each canton, emails and flyers were sent to the directors of all special schools (*n* = 54) for students with IDD. Among them, 36 special schools' directors (66.67%) gave their approval and therefore were invited to share the information with their staff. The recruitment materials briefly outlined the purpose of the study and directed voluntary participants to an online survey.

To be eligible to complete the online survey, respondents had to meet the following criteria: (1) work in a special school for students with IDD, (2) work with students aged 4 to 18 years old, (3) be assigned to a specific classroom (i.e., not part of the itinerant staff rotating between classes) and (4) be either a special education teacher or a social worker in education. In special schools for students with IDD in Switzerland, the organisation of classroom roles varies depending on the region, the school and the individual classroom. Special education teachers and social workers in education often work collaboratively in the same classroom; however, they are not necessarily present on the same days (part‐time employment). They often teach alone most of the days of the week in classes of three to eight students (*M* = 5) with IDD, seconded by a classroom aide. In some classes, both professionals are present together one to 4 days per week (Meuli 2024). Special education teachers generally retain primary responsibility for instruction and curriculum delivery, while social workers in education mainly provide support to facilitate students' participation in daily routines and learning activities. However, in practice, qualifications and roles are not always clearly differentiated. In the French‐speaking region of Switzerland, many professionals employed as special education teachers in special schools for students with IDD do not have formal training in special education but instead hold a bachelor's degree in social work (Meuli 2024).

A total of 239 respondents logged in to complete the online survey. Of these, 22 logged in but did not provide any usable data, and 12 did not meet one or more of the inclusion criteria. As a result, 205 respondents (85.77%) were retained. Among them, half (50.73%) were social workers in education, and the other half (49.27%) were special education teachers. Respondents reported working with an average of 6.01 students per class (SD = 2.54), of whom an average of 2.88 (SD = 2.08) reportedly displayed CB. The average number of staff members per class was 2.74 (SD = 1.36), corresponding to a mean student‐to‐adult ratio of 2.49 students per adult (SD = 1.70). Only a third of the respondents (34%) stated that they had received adequate training on functional assessment and even less (22%) on applied behaviour analysis in relation to CB. Please refer to Table 1 for further details on the respondents.

**TABLE 1:** Description of respondents working as staff in special schools (*n* = 205).

	*n*	%
Role		
Social worker in education	104	50.73%
Special education teacher	101	49.27%
Canton		
Freiburg	38	18.54%
Geneva	38	18.54%
Jura	32	15.61%
Neuchâtel	32	15.61%
Valais	10	4.88%
Vaud	55	26.83%
Diploma		
Master's	90	43.90%
Bachelor	94	45.85%
Post‐secondary	19	9.27%
Missing	2	0.98%
Formation		
In training	32	15.61%
Graduated	173	84.39%
Years of experience		
1 to 2 years	40	19.51%
3 to 5 years	50	24.39%
6 to 10 years	39	19.02%
11 to 15 years	30	14.63%
More than 15 years	46	22.44%
Students per classroom		
3 students or less	14	6.83%
4 to 5 students	94	45.85%
6 to 7 students	40	19.51%
8 to 9 students	26	12.68%
10 to 11 students	24	11.71%
12 or more students	7	3.41%
Students with challenging behaviour		
No students	19	9.27%
1 to 2 students	75	36.59%
3 to 4 students	75	36.59%
5 to 6 students	19	9.27%
7 to 8 students	9	4.39%
9 or more students	8	3.90%

### 3.2 Measures

The online survey included a respondent sociodemographic section, followed by questions about classroom characteristics and students' CB. This questionnaire was developed specifically for the present study and is not a standardised, norm‐referenced measure.

#### 3.2.1 Sociodemographic

Respondents were asked about their role in the classroom (teacher or educator), canton (i.e., a state‐level administrative region), highest educational qualification, years of experience and the number of students in their classroom. Participants were then presented with the following definition: “In this survey, CB are defined as intense behaviours that raise concern among professionals. They disrupt learning, pose a risk to the student or to others, cause physical harm, damage property, or seriously interfere with the teaching and learning process. CB are displayed frequently by the student and are difficult to manage”. They were then asked to report how many students in their classroom displayed behaviours consistent with this definition. For each of the student identified as displaying CB, respondents were asked to provide information on age, diagnosis and communication (student need for an alternative communication system to be able to communicate functionally in everyday life).

#### 3.2.2 Challenging Behaviours

This section was completed for each student identified as displaying CB. Respondents were presented with six types of CB, each accompanied by a brief definition and examples: (1) oppositional behaviour (e.g., refusing to follow instructions, such as not participating in activities, challenging authority or deliberately ignoring rules), (2) property destruction (damaging property, such as intentionally breaking windows, tearing books or other materials or damaging classroom equipment), (3) physical aggression (physically attacking another person, such as hitting, kicking or fighting), (4) verbal aggression (verbally attacking another person, such as provoking, challenging, insulting or threatening), (5) self‐injurious behaviour (causing harm to oneself, such as hitting one's head or biting oneself) and (6) socially inappropriate behaviour (engaging in behaviours that are not socially acceptable, such as shouting loudly, making offensive gestures or exposing one's private parts). For each identified student with CB, respondents were asked to indicate the frequency with which the student exhibited each of the six types of CB using a 5‐point Likert scale (1 = *never*, 2 = *monthly*, 3 = *weekly*, 4 = *daily*, 5 = *hourly*). Responses were not mutually exclusive, such that a given student could be reported as exhibiting multiple types of behaviour at different frequencies.

### 3.3 Participants

A total of 645 individual students' profiles were reported by the respondents. However, 10 student's profiles (1.6%) contained missing data that rendered them unsuitable for the main analyses, leaving a final sample of 635 students included in the study. Participants were age in average 10.87 year‐old (SD = 3.88), with the youngest being 4 and the oldest being 18 years of age. Regarding diagnoses, 451 students (71.02%) had autism, 395 (62.21%) had an intellectual disability, 93 (14.65%) had attention‐deficit/hyperactivity disorder, 13 (2.05%) had another type of diagnosis (e.g., chromosomal syndrome, physical impairment, psychiatric disorder) and 10 (1.57%) were undiagnosed. For a more detailed account using mutually exclusive diagnostic groupings, please refer to Table 2. Among participants, 415 (65.36%) required alternative communication systems.

**TABLE 2:** Characteristics of participating students in special schools (*n* = 635).

		*n*	%	*M*	SD	*Mdn*	Mode
Age (in years)				10.87	3.88		
Alternative communication		415	65.35				
Diagnosis							
Autism an ID		216	34.02%				
Autism only		178	28.03%				
ID only		125	19.69%				
Autism and ADHD		21	3.31%				
ID and ADHD		18	2.83%				
ADHD only		18	2.83%				
Autism, ID and ADHD		36	5.67%				
Other diagnostics		13	2.05%				
No diagnostics		10	1.57%				
Challenging behaviours							
Opposition		592	93.23	3.68	1.10	4	4
Destruction		348	54.8	2.13	1.25	2	1
Physical aggression		393	61.89	2.28	1.23	2	1
Verbal aggression		218	34.33	1.76	1.19	1	1
Self‐injurious		217	34.17	1.75	1.17	1	1
Socially inappropriate		569	89.61	3.33	1.17	4	4

*Note:* Challenging behaviours scores range from 1 (*never*) to 5 (*hourly*).

Abbreviations: ADHD = attention‐deficit/hyperactivity disorder, ID = intellectual disability.

### 3.4 Analyses

Analyses were conducted using JASP (v. 0.19.1) and R Studio (v. 2024.12.1). To address the first objective, descriptive statistics (frequencies, means, standard deviations, medians and modes) were calculated to describe the prevalence and frequency of each type of CB among students in special schools, as reported by staff.

To address the second objective, inter‐class correlation coefficients were first calculated using the *lme4* packages (Bates et al. 2015) and indicated substantial clustering of student behaviours within respondents across the six variables (ranging from 0.16 to 0.40), suggesting that the nesting of data could not be ignored. To account for this nesting, a multilevel latent class analysis was conducted using the *multiLCA* package (Lyrvall et al. 2025). As the package requires binary variables, ordinal behaviour ratings were dichotomised (0 = *never* or *monthly*; 1 = *weekly, daily* or *hourly*) in order to run the analysis. Inter‐class correlation coefficients calculated on the dichotomised data remained notable (0.11 to 0.37), further justifying a multilevel approach.

To address the third objective, a multivariate analysis of variance (MANOVA) tested group differences across variables. Please note that the original ordinal behaviour ratings were retained when describing the groups to provide a more detailed overview. Where significant effects were found, one‐way ANOVAs were conducted for individual variables. Shapiro–Wilk tests assessed normality. Where assumptions were violated, Kruskal–Wallis tests were used for continuous variables and chi‐squared tests for categorical ones. Dunn's post hoc tests with Bonferroni correction were applied following significant Kruskal–Wallis results, and standardised residuals (adjusted Pearson) were examined to interpret differences in categorical variables.

## 4 Results

### 4.1 Types and Frequency of CB Displayed by Students According to Staff

School staff reported that, among students in their classrooms (*M* = 6.02, SD = 2.54), about half of them displayed persistent CB (*M* = 2.88, SD = 2.08). Most students considered as displaying CB exhibited oppositional behaviour (93.23%) and socially unacceptable behaviours (89.61%) daily (median = 4). Physical aggression (61.89%) and destructive behaviour (54.80%) were reported in over half of the students, with both occurring monthly (median = 2). Verbal aggression (34.33%) and self‐injurious behaviours (34.17%) were the least frequently observed, with approximately one third of students displaying these behaviours, typically never (median = 1), according to respondents. A detailed description of students' profiles is provided in Table 2.

### 4.2 Profiles Among Students With IDD

Multilevel latent class analysis was used to assess the fit of 18 models specifying student classes at level 1 and respondent groups at level 2. Two solutions emerged as optimal: a 3(student) × 3(respondent) model and a 4(student) × 2(respondent) model. The 3 × 3 model provided a marginally better statistical fit (BIC = 3923; ICL = 4122; AIC = 3836) but had relatively low entropy (*R* ^2^ entropy = 0.53–0.55). In contrast, the 4 × 2 model showed slightly worse fit (BIC = 3940; ICL = 4023; AIC = 3837) but substantially higher entropy (*R* ^2^ entropy = 0.67–0.70), indicating clearer class separation. Given our aim to maximise interpretability and classification quality, we retained the 4 × 2 model despite a marginally weaker statistical fit, and group membership was integrated into the main dataset. Figure 1 depicts the outcomes of the selected model, while Figure 2 depicts the probability of a student in each of the groups to display each type of behaviours. At the respondent level, two types were identified: the first (69.8% of respondents) was mainly associated with student in Group 1 and 3, while the second (30.2% of respondents) was more strongly linked to student in Group 2 and 4.

**FIGURE 1:**
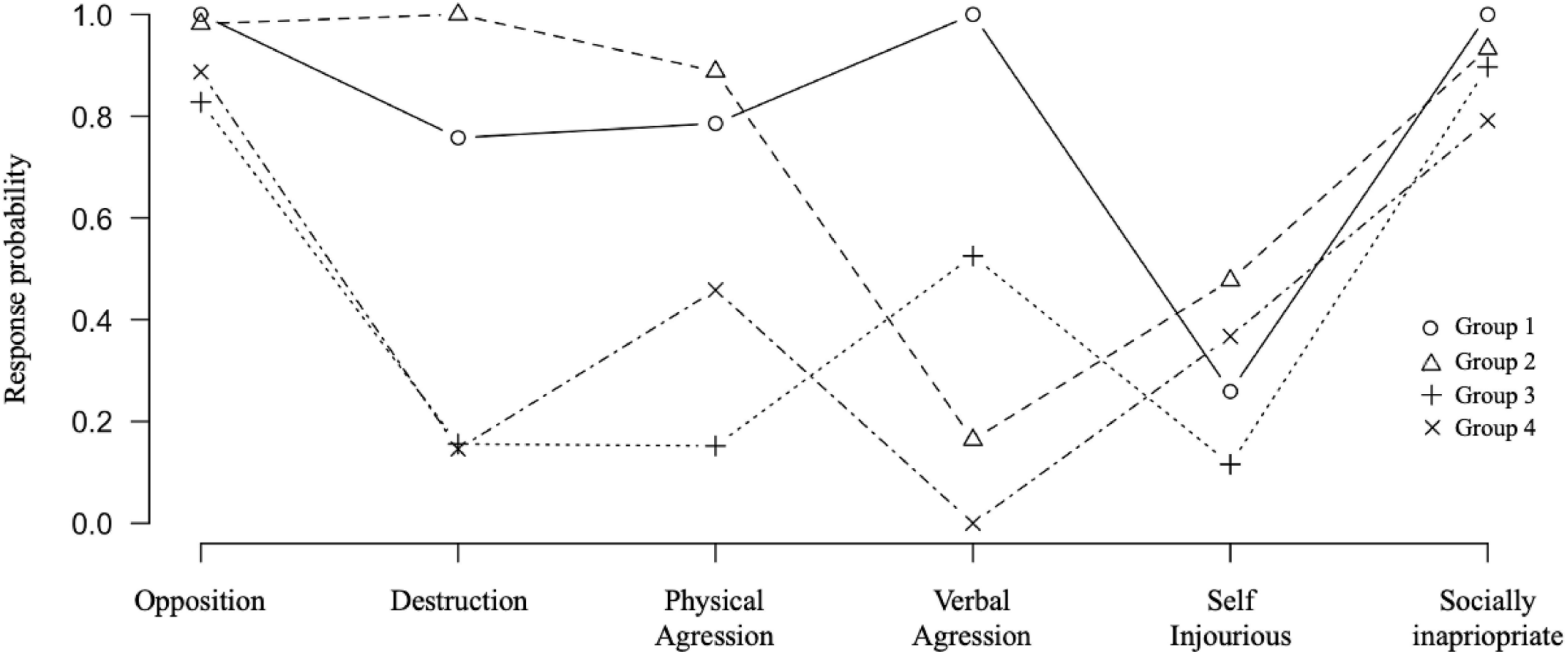
Probability of response per cluster on each of the six behavioural variables.

**FIGURE 2:**
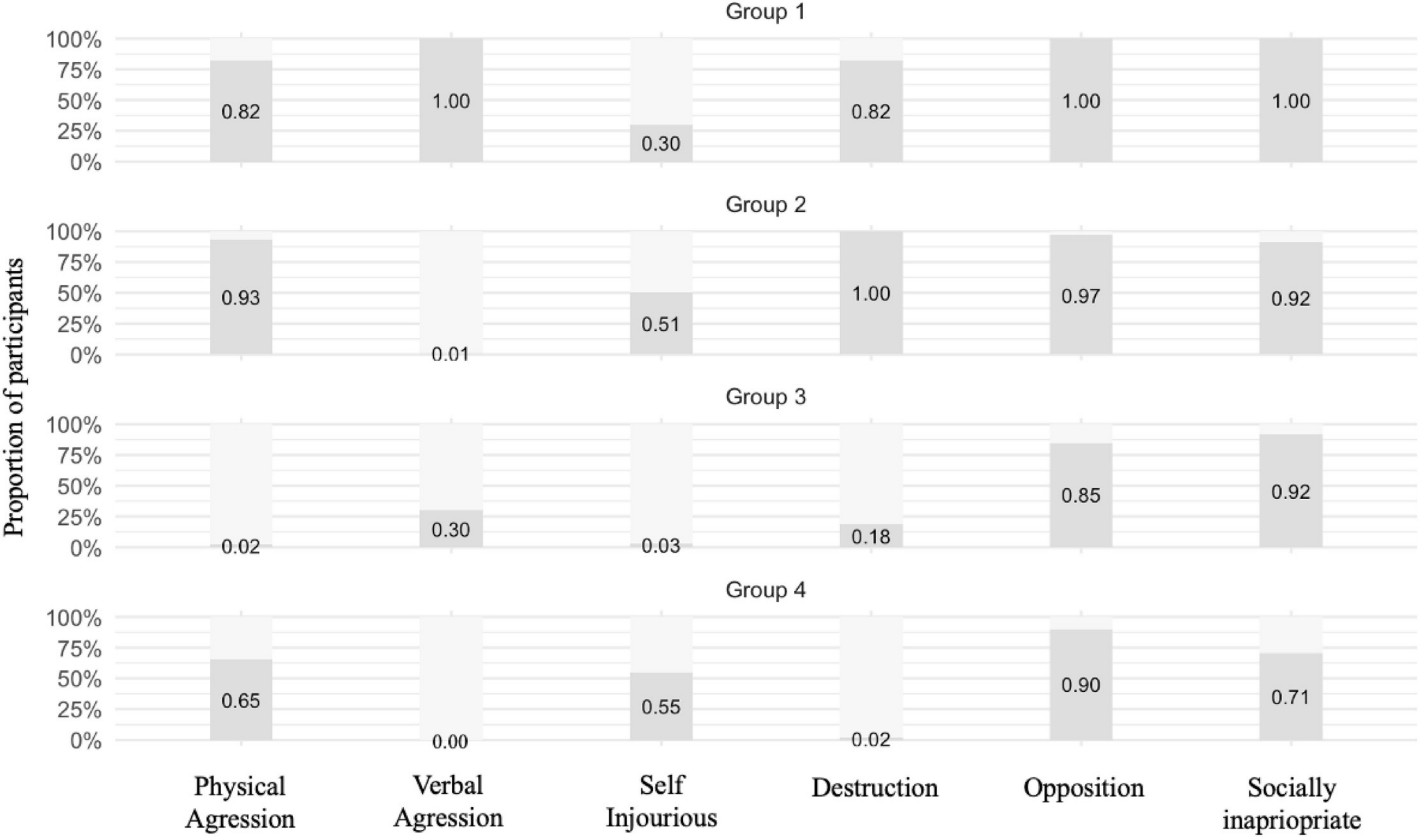
Proportion of participants exhibiting challenging behaviours at least once a week across six behaviour categories and four groups.

#### 4.2.1 Group 1—Overall High CB (*N* = 169, 27%)

All students in this group (100%) displayed oppositional behaviour and socially inappropriate behaviour daily (*Mdn* = 4) and verbal aggression mostly on a weekly basis (*Mdn* = 3). Physical aggression and destruction were both slightly less common, with 82% of students displaying them mostly on a weekly basis (*Mdn* = 3). Self‐harming behaviour was present in only 30% of students (*Mdn* = 1, *never*). Diagnostically, about half had an intellectual disability (51.5%) or autism (56.2%), and about a quarter had attention‐deficit/hyperactivity disorder (26.0%). Among them, 42.6% required an alternative communication system. The mean age was 11 years (SD = 3.57).

#### 4.2.2 Group 2—High Aggressivity and Destruction (*N* = 200, 32%)

All students in this group (100%) displayed destructive behaviour daily (*Mdn* = 3). Most also displayed oppositional behaviour (97%) and socially inappropriate behaviour (92%) on a daily basis (*Mdn* = 4). Physical aggression was displayed by 83% on a daily basis (*Mdn* = 3), while self‐harm was present in 46% (*Mdn* = 1, *never*). Verbal aggression was almost non‐existent (1%; *Mdn* = 1, *never*). In terms of diagnoses, 64.0% had intellectual disability, 85.0% autism and 10.5% attention‐deficit/hyperactivity disorder. A majority (86.5%) required an alternative communication system. The average age was 9.90 years (SD = 3.98).

#### 4.2.3 Group 3—Overall Low CB (*N* = 131, 22%)

Most students in this group displayed oppositional behaviour (82%) and socially inappropriate behaviour (95%), but mostly on a weekly basis (*Mdn* = 3). About a third (36%) displayed verbal aggression (*Mdn* = 1, *never*). It is noteworthy that self‐harm (4%), destruction (2%) and physical aggression (2%) were almost non‐existent (*Mdn* = 1, *never*). Regarding diagnoses, 64.9% had intellectual disability, 61.1% autism and 16.8% attention‐deficit/hyperactivity disorder. About half (45.8%) required an alternative communication system. The group's mean age was 12.17 years (SD = 3.64).

#### 4.2.4 Group 4—Physical Aggression and Self‐Harm (*N* = 135, 21%)

Most students in this group displayed oppositional behaviour (90%) on a daily basis (*Mdn* = 4). About two‐thirds (68%) displayed socially inappropriate behaviour on a weekly basis (*Mdn* = 3). Physical aggression (63%) and self‐harm (52%) were both displayed monthly (*Mdn* = 2). Destruction (5%) was very rare, and verbal aggression was absent (*Mdn* = 1, *never*). Diagnostically, 70.4% had intellectual disability, 78.5% autism and 4.4% attention‐deficit/hyperactivity disorder. A majority (81.5%) required an alternative communication system. The group's mean age was 10.86 years (SD = 3.99).

### 4.3 Differences in Behavioural Profiles Regarding CB, Age, Diagnoses and Communication Skills

The four groups were first compared using a MANOVA, which indicated significant differences across all behavioural variables (*F* (3) = 43.03, *p* < 0.001). However, both Mardia's test for multivariate normality (*χ* ^*2*^ = 1203(45), *p* < 0.001) and the Shapiro–Wilk test for univariate normality (*W* = 0.915, *p* < 0.001) revealed violations of the normality assumption. Consequently, to further explore differences at the individual variable level, a series of nonparametric Kruskal–Wallis tests were conducted. These tests confirmed significant differences across all behavioural variables (all *p* < 0.001). Full details are provided in Table 3, which presents the mean scores and standard deviations for each group across all behavioural variables, along with Kruskal–Wallis test results, effect sizes and significant pairwise comparisons based on Dunn's post hoc tests with Bonferroni correction. To determine whether the behavioural profiles differed in terms of age, diagnoses and communication skills, a similar analytical approach was applied. The results of these analyses are presented in Table 4.

**TABLE 3:** Descriptive statistics and differences per group on the behavioural variables used for clustering.

	Group 1 (*n* = 169)		Group 2 (*n* = 200)		Group 3 (*n* = 131)		Group 4 (*n* = 135)		Kruskal–Wallis test			
	*M*	SD	*M*	SD	*M*	SD	*M*	SD	H (df = 3)	*p*	*ε*2	Differences
Opposition	4.01	0.76	3.99	0.96	3.06	1.28	3.43	1.14	67.03	< 0.001	0.106	3 < 4 < 1,2
Destruction	2.63	1.14	3.13	0.98	1.02	0.12	1.11	0.54	425.1	< 0.001	0.671	3,4 < 1 < 2
Physical aggression	2.57	1.04	2.91	1.23	1.02	0.15	2.19	1.14	222	< 0.001	0.35	3 < 4 < 1 < 2
Verbal aggression	3.33	0.92	1.01	0.16	1.66	1.04	1.00	0.00	490	< 0.001	0.773	2,4 < 3 < 1
Self‐injurious	1.64	1.14	2.05	1.31	1.07	0.38	2.08	1.22	82.15	< 0.001	0.130	3 < 1 < 2,4
Socially inappropriate	3.64	0.95	3.58	1.11	3.28	1.01	2.64	1.36	45.28	< 0.001	0.086	1,2 < 3 < 4

*Note:* Significant differences were identified using Dunn's post hoc test with Bonferroni correction.

**TABLE 4:** Descriptive statistics and differences for the four groups.

	Group 1		Group 2		Group 3		Group 4		Chi‐squared test		
	*n*	%	*n*	%	*n*	%	*n*	%	χ2(df = 3)	*p*	Differences
Age in years (*M*, SD)	11.00	3.57	9.90	3.98	12.17	3.64	10.86	3.99	9.454	< 0.001	2 < 1,4 < 3
Alternative communication	72	42.60%	173	86.50%	60	45.80%	110	81.48%	115.8	< 0.001	1 < 3 < 4 < 2
Intellectual disability	87	51.48%	128	64.00%	85	64.89%	95	70.37%	12.77	0.005	1 < 2,3 < 4
Autism spectrum disorder	95	56.21%	170	85.00%	80	61.07%	106	78.52%	46.99	< 0.001	1 < 3 < 4 < 2
Attention‐deficit/hyperactivity disorder	44	26.04%	21	10.50%	22	16.79%	6	4.44%	32.01	< 0.001	2,4 < 3 < 1

*Note:* For age (continuous variable), a Kruskal–Wallis test was used, and the reported variable is an H with the *p*‐value adjusted for a family of four. Significant differences were identified using standardised pearson residuals.

## 5 Discussion

This study aimed to enrich our understanding of CB exhibited by students with IDD in special education classrooms. An online survey completed by 239 school staff members provided data on 635 students. These data allowed us to describe the types of CB encountered in special schools in Switzerland's French‐speaking regions. Through multilevel latent class analysis, we identified four distinct profiles, each characterised by different patterns and frequencies of CB. These profiles offer valuable insights into the heterogeneity of behavioural presentations among students with IDD. This underscores the need for adapted interventions, comprehensive staff training and support structures for school staff. Respondents reported that approximately half of the students in their classrooms displayed persistent CB, with the vast majority of these students exhibiting oppositional and socially inappropriate behaviours daily.

These findings highlight the importance of implementing comprehensive, school‐wide strategies capable of supporting all students, particularly those with persistent or severe CB. Evidence from this study reinforces the value of PBS as a structured, evidence‐based, multi‐tiered framework that facilitates student‐centred interventions through close collaboration with key stakeholders to achieve meaningful outcomes in reducing CB (Gore et al. 2013; Kincaid 2018). It emphasises best practices such as functional behaviour assessment, preventive strategies, teaching of alternative behaviours and positive reinforcement (Beqiraj et al. 2022; Cooper et al. 2020; Dunlap et al. 2018). By organising supports across three tiers (universal strategies for all students, targeted interventions for students at higher risk and individualised support for students with severe needs), PBS provides a scalable framework for addressing CB in schools (Corcoran and Edward Thomas 2021). Importantly, the present study contributes context‐specific evidence regarding the types and frequency of CB in special schools in French‐speaking Switzerland, highlighting the need for frameworks that are both aligned with best practices and adapted to the realities, resources and needs of schools, staff and students.

### 5.1 CB in the Context of French‐Speaking Switzerland Special Schools

One such context‐specific feature of this study was the age range of participating students (4 to 19 years), which reflects a particular feature of the Swiss education system. While many international studies on school‐aged students with IDD typically focus on participants aged between 4 and 12 years (see Anixt et al. 2020; Iovannone et al. 2009), the mean age in our sample was 10.78 years (SD = 3.88), with a range extending from 4 to 19 years. This broader age span is attributable to Swiss legislation, which guarantees access to schooling for individuals with special educational needs until the age of 19 or even up to 21 in some cases (*Loi fédérale sur l'élimination des inégalités frappant les personnes handicapées*, 2004). This influences the composition of the student population in special schools and should be taken into account when interpreting findings or comparing with studies from other countries.

Regarding CB, the most frequently reported type by school staff was opposition, observed in 93% of students described as displaying persistent CB. This figure is particularly striking given that the reported frequency (*Mdn* = 4) suggests that oppositional behaviours occur daily in most cases. A similar pattern emerged for socially inappropriate behaviour, which was reported in 90% of students displaying persistent CB, also with a median frequency of 4, indicating daily occurrence. Prior research has generally reported physical aggression and property destruction as the most prevalent forms of CB, followed by self‐injurious and socially inappropriate behaviours, with oppositional behaviour mentioned less frequently, both in parental reports from home (Esteves et al. 2021) and teacher reports from school settings (Nicholls et al. 2020). We hypothesise that the discrepancy with Esteves et al. (2021) may be due to the school‐based nature of our study, where oppositional and socially inappropriate behaviours are likely to be perceived as especially disruptive to classroom functioning, and thus more salient to school staff than to parents. In contrast, the divergence from the findings of Nicholls et al. (2020) may be attributed to their use of a modified version of the Behaviour Problems Inventory—Short Form (Rojahn et al. 2012), which does not include subscales for socially inappropriate and oppositional behaviours, but rather a subscale for stereotyped behaviours, which was rated lower than the others. On their end, Amstad and Müller et al. (2020), who investigated teachers' perceptions of stress associated with student behaviour in special schools in the German‐speaking part of Switzerland, found that physically aggressive and self‐injurious behaviours were perceived as the most stressful. Since the authors did not specifically report the frequency of these behaviours, comparisons with our findings are not possible. However, it is worth noting that in our sample, physical aggression was reported to be present in 62% of students considered as displaying CB, while self‐injurious behaviour was reported in 34%.

### 5.2 Behavioural Grouping

The identification of four distinct behavioural groups presented in this article highlights the diverse nature of CB among students with IDD, which aligns with previous research emphasising the multifaceted character of CB in this population (Bowring et al. 2016; Reyes‐Martín et al. 2022) and reinforces the importance of tailored intervention approaches. Also consistent with prior research, profiles characterised by higher frequency and severity of CB were more prevalent among younger students and those with autism and greater communication needs (Fodstad et al. 2012; Hastings et al. 2013; Nicholls et al. 2020). This grouping represents an effort to categorise students' behavioural patterns and support needs within the three tiers of PBS (universal, targeted and intensive), with each profile reflecting distinct patterns of behavioural risk and corresponding support needs. Indeed, the profiles identified in this study can be conceptualised along a continuum of intensity and individualisation of support (Simonsen et al. 2021), while recognising that Tier 1 practices remain foundational for all students (Simonsen et al. 2020).

Students in Group 1 (Overall High CB) and Group 2 (High Aggressivity and Destruction) shared similarly elevated levels of oppositional behaviour, socially inappropriate behaviour and aggression. However, they differed in their communication profiles and behavioural topographies.

Students in Group 1 predominantly displayed verbal aggression while showing low levels of self‐injury. This group was characterised by the lowest need for alternative communication systems, as well as a lower prevalence of intellectual disability and autism, suggesting stronger communication capacity and thus explaining the higher presence of verbal aggression. The high prevalence of attention‐deficit/hyperactivity disorder in this group, combined with a lower reliance on alternative communication systems, may be linked to impulsivity and difficulties with behavioural inhibition despite relatively strong communication skills. In other words, this pattern suggests difficulties related more to behavioural regulation, impulsivity and performance deficits than to fundamental communication access. For students in this group, Tier 1 PBS strategies are likely to be particularly relevant. Examples include clear and consistently taught classroom expectations, frequent opportunities to respond, predictable routines throughout the day and high rates of behaviour‐specific praise (Alter and Haydon 2017; Floress et al. 2017). This could help create a structured environment that prevents escalation and supports all students (Simonsen et al. 2020). Tier 2 interventions may also be needed, including structured self‐regulation instruction, small‐group social skills training or check‐in/check‐out systems providing regular feedback and reinforcement (Hawken et al. 2020). These strategies align with PBS guidance on intensifying core practices rather than immediately moving to highly individualised interventions, and they directly address the oppositional and verbally aggressive behaviours observed in this group.

In contrast, students in Group 2 exhibited virtually no verbal aggression but markedly higher levels of self‐injurious behaviour. They were significantly younger, showed the highest reliance on alternative communication systems and had the greatest prevalence of autism. Notably, this was also the largest group, which may reflect the broader behavioural and support profiles commonly observed in special school settings. This combination of severe CB and substantial communication needs suggests that these students may require intensive Tier 3 PBS interventions. Such interventions include function‐based approaches informed by functional behaviour assessment, with a strong emphasis on functional communication training, proactive environmental adaptations (e.g., visual supports, structured transitions, reduced sensory load) and explicit instruction in alternative behaviours (Dunlap et al. 2018; Simonsen et al. 2021). Although resource‐intensive, these interventions may be particularly effective in addressing highly disruptive and aggressive behaviours, while also providing staff with the structured and consistent support required to manage these challenges (Flynn et al. 2019). Moreover, underlying communication difficulties may contribute to the escalation of CB (Sigafoos et al. 2016), suggesting that the systematic development and use of communication supports can enhance communicative competence and, in turn, reduce CB (Hetzroni 2009). This group shows the highest frequency of self‐injurious behaviours among the four profiles. Evidence indicates that intensive behavioural interventions can substantially reduce these behaviours, with outcomes largely consistent across participant characteristics, supporting the broad applicability of evidence‐based approaches (Moore et al. 2024; Morano et al. 2017). However, effectiveness is strongly influenced by the implementer and setting, underscoring the need for early, intensive Tier 3 interventions that are sustainably embedded within educational contexts for students in this group.

Students in Groups 3 (Overall Low CB) and 4 (Physical Aggression and Self‐Harm) presented comparatively lower overall frequencies of CB but differed in the presence of self‐injury, verbal aggression and socially inappropriate behaviour. Compared to Groups 1 and 2, both profiles reflect patterns of frequent but generally less dangerous behaviours that may nonetheless place considerable strain on special school staff.

Students in Group 3 displayed an overall low frequency of CB. While socially inappropriate and oppositional behaviours were present, the latter occurred significantly less often than in the other groups. More severe behaviours such as destruction, physical aggression and self‐injurious behaviour were rarely reported and were less frequent than in other groups. Combined with the fact that these students were older on average, this may suggest a more stable behavioural profile. Although less severe, this profile may still be disruptive in social and classroom contexts without necessarily involving physical harm to self or others. Students in this group may respond well to Tier 1 interventions, with Tier 2 support considered if further needs arise or if initial measures are not effective. Strategies such as implementing a more structured daily routine, clarifying expectations and providing explicit instruction on appropriate classroom behaviour, or offering students meaningful choices can effectively decrease CB and reduce oppositional and socially inappropriate behaviours (McCormick et al. 2003; Simonsen et al. 2020). Nonetheless, low but persistent levels of socially inappropriate behaviours indicate the importance of maintaining explicit instruction on social rules and expectations.

Group 4 was characterised by very low rates of destruction and verbal aggression, as well as the lowest levels of socially inappropriate behaviour compared to other groups. However, students displayed high levels of self‐injurious behaviour and some moderate physical aggression. A large proportion of these students used alternative communication, and rates of intellectual disability were the highest across groups, while autism spectrum disorder was the second most prevalent diagnosis. This profile suggests vulnerabilities related to emotion regulation, sensory processing and limited communicative means. Students presenting this pattern may therefore benefit from sustained Tier 2 PBS interventions targeting social skills, emotional regulation and functional communication, with supports embedded within daily classroom routines (Hawken et al. 2020; McDaid et al. 2022). If behaviours persist or pose safety risks, Tier 3 interventions may be warranted, involving function‐based approaches informed by functional behaviour assessment and a strong emphasis on functional communication training and proactive environmental adaptations (e.g., visual supports, structured transitions, reduced sensory load; Dunlap et al. 2018; Simonsen et al. 2021). This is particularly relevant for self‐injurious behaviours, as students in this group present high frequencies of such behaviours and are therefore likely to require early, intensive Tier 3 interventions, with a strong emphasis on high‐quality implementation within everyday educational contexts (Moore et al. 2024; Morano et al. 2017).

Taken together, these findings indicate that PBS functions as a flexible, tiered framework that can be meaningfully aligned with empirically derived CB profiles in special school contexts. By linking distinct behavioural patterns to potentially relevant PBS tiers and intervention strategies, this study provides context‐specific evidence to inform PBS implementation in French‐speaking Swiss special schools and supports its use as a coherent approach for matching intervention intensity to students' behavioural and communicative profiles.

### 5.3 Limitations

A first limitation of the present study is the fact that students' gender was not included as a variable in the questionnaire. This prevents the assessment of potential gender differences between groups and how they relate to frequency and types of CB. Gender can influence behavioural patterns (Graziosi and Perry 2023), and the absence of such data may restrict the comprehensiveness of the findings. A second limitation is the reliance on self‐reported data provided by school staff. Although we attempted to mitigate this by providing descriptions and examples for each type of CB, staff perceptions and interpretations may have introduced bias. A third limitation is the nonnormal distribution of data, although common in behavioural research in special schools. This required the use of nonparametric methods, which, while appropriate, may have reduced statistical power. However, consistent findings across tests support the robustness of the results. A fourth limitation is the lack of clear information on students' diagnoses, which was not always available to school staff. Findings related to diagnosis should therefore be considered with caution. Finally, because the primary aim of this study was to examine student characteristics, the identification of two respondent groups reflects only the nesting structure used in the analysis rather than a substantive focus on school staff.

## 6 Conclusion

The detailed description of the types and frequency of CB, along with the identification of distinct behavioural profiles among students with IDD in special schools offers valuable insights into the specific needs of this population. These findings can be used to help inform the development of targeted, evidence‐based interventions to improve the educational experiences and outcomes for students with IDD. Future research should focus on how to provide tailored training and ongoing support for special school staff, helping them implement effective CB management programmes that address the unique needs of the students they work with. Additionally, future studies with specific research questions could examine how variables such as class size, student‐to‐staff ratios and teacher experience impact teacher perceptions of CBs, using a modelling approach that takes into account the characteristics of school staff.

## Funding

This work was supported by the Swiss National Science Foundation (100019‐227842).

## Disclosure

No generative AI was used for the scientific content, drafting, analysis or interpretation of this manuscript. In accordance with this journal's guidelines, which state that language editing tools are outside the scope of the AI Use Declaration, AI was used solely to improve spelling and grammar.

## Ethics Statement

Given its survey‐based design, the present study was reviewed and deemed exempt from formal ethics approval by the Swiss Commission for Ethics in Human Research of the Canton of Vaud (reference number: Req‐2024‐00420).

## Conflicts of Interest

The authors declare no conflicts of interest.

## Data Availability

This study's data is not publicly available. However, data may be available upon request, subject to institutional ethics approval and data protection regulations.
